# Supplementary material for: Determination of topological edge quantum numbers of fractional quantum Hall phases by thermal conductance measurements
Source: Nat Commun. 2022 Sep 3;13:5185. doi: 10.1038/s41467-022-32956-z (PMC9440925; doi:10.1038/s41467-022-32956-z)
Supplement: Supplementary file 1 — Supplementary Information [file 41467_2022_32956_MOESM1_ESM.pdf]

Supplementary Information for "Determination of topological edge quantum numbers of fractional quantum Hall phases by thermal conductance measurements"

Saurabh Kumar Srivastav[1], Ravi Kumar[1], Christian Spånslätt[2], K. Watanabe[3], T. Taniguchi[3], Alexander D. Mirlin[4,5,6,7], Yuval Gefen[4,8], and Anindya Das[1*]

[1]*Department of Physics, Indian Institute of Science, Bangalore, 560012, India.*

[2]*Department of Microtechnology and Nanoscience (MC2),Chalmers University of Technology, S-412 96 Göteborg, Sweden.*

[3]*National Institute of Material Science, 1-1 Namiki, Tsukuba 305-0044, Japan.*

[4]*Institute for Quantum Materials and Technologies, Karlsruhe Institute of Technology, 76021 Karlsruhe, Germany.*

[5]*Institut für Theorie der Kondensierten Materie, Karlsruhe Institute of Technology, 76128 Karlsruhe, Germany.*

[6]*Petersburg Nuclear Physics Institute, 188300 St. Petersburg, Russia.*

[7]*L. D. Landau Institute for Theoretical Physics RAS, 119334 Moscow, Russia.*

[8]*Department of Condensed Matter Physics, Weizmann Institute of Science, Rehovot 76100, Israel.*

[*]anindya@iisc.ac.in

Supplementary Note 1: Device fabrication, characterization, and noise measurement setup

In order to observe well-developed fractional quantum Hall states in graphene, we have used a graphite gated hexagonal boron nitride (hBN) encapsulated graphene (SLG) device (hBN/SLG/hBN/graphite). For the fabrication of the device, we have followed the standard dry transfer pick-up technique [1]. It involves the mechanical exfoliation of hBN and graphite crystals on a oxidized silicon wafer using scotch tape. First, a clean blister free hBN layer of thickness of $\sim$ 25 nm was picked up at 90°C using a Poly-Bisphenol-A-Carbonate (PC) coated Polydimethylsiloxane (PDMS) stamp placed on a glass slide, attached to tip of a home build micromanipulator. This hBN flake was aligned on top of previously exfoliated graphene. The graphene was picked up at 90°C. The next step involved the pick up of bottom hBN ($\sim$ 25 nm). This bottom hBN was picked up using the previously picked-up hBN/SLG following the previous process. The hBN/SLG/hBN heterostructure was used to pick-up a graphite flake following the previous step. Finally, the resulting hetrostructure (hBN/SLG/hBN/graphite) was dropped on top of an oxidized silicon wafer of thickness 285 nm at temperature 180°C. To remove the residues of PC, the final stack was cleaned in chloroform ($CHCl_3$) overnight followed by cleaning in acetone and iso-propyl alcohol (IPA). To get the region free from any bubbles and residues, we further performed atomic force microscopy (AFM) topography of the flake. After this, Poly-methyl-methacrylate (PMMA) was coated on the heterostructure to define the contact regions in Hall probe geometry using electron beam lithography (EBL) in clean area of the stack. Apart from the conventional Hall probe geometry, we defined a region of $\sim$ 5.5 μm^2 area in the middle of the heterostructure, which acts as floating metallic reservoir. After EBL, reactive ion etching (mixture of CHF_3 and O_2 gas with flow rate of 40 sccm and 4 sccm, respectively at 25°C with RF power of 60W) was used to define the edge contacts. The etching time was optimized such that the bottom hBN did not etch completely to isolate the contacts from the bottom graphite flake, which was used as a back gate. Finally, thermal deposition of Cr/Pd/Au (3/12/60 nm) was done in an evaporator chamber having a base pressure of $\sim 1 - 2 \times 10^{-7}$ mbar. After deposition, a lift-off procedure was performed in hot acetone and IPA. This resulted in a Hall bar device along with the floating metallic reservoir connected to the both sides of SLG by the edge contacts. The AFM topography of stack and the optical image of the full device is shown in Supplementary Fig. 1(a) and Supplementary Fig. 1(b), respectively. The distances from the floating contact to the ground contacts were $\sim 5\mu m$. All the measurements were done in a cryo-free dilution refrigerator having a base temperature of $\sim$ 20mK. The electrical conductance was measured using the standard lock-in technique whereas the thermal conductance was measured employing noise thermometry based on an LCR resonant circuit at resonance frequency of $\sim$ 740kHz and amplified by a home made preamplifier at 4K followed by room temperature amplifier, and finally measured by a spectrum analyzer.

Total two-terminal resistances (R) of the device was measured as a function of the bottom graphite

gate voltage (V_{BG}) at zero magnetic field. The measured data is fitted with the equation[2–5]

$$R = R_C + \frac{L}{We\mu\sqrt{(n_0^2 + (\frac{C_{BG}(V_{BG}-V_{DP})}{e})^2)}}\,, \tag{1}$$

where R_C, L, W, μ, and e are, respectively, the contact resistance, length, width, mobility, and electron charge. The carrier concentration of the channel is given by $\frac{C_{BG}(V_{BG}-V_{DP})}{e}$ with C_{BG} and V_{DP} being the capacitance per unit area of the bottom graphite gate, and the voltage at the charge neutrality point, respectively. n_0 is the charge inhomogeneity.

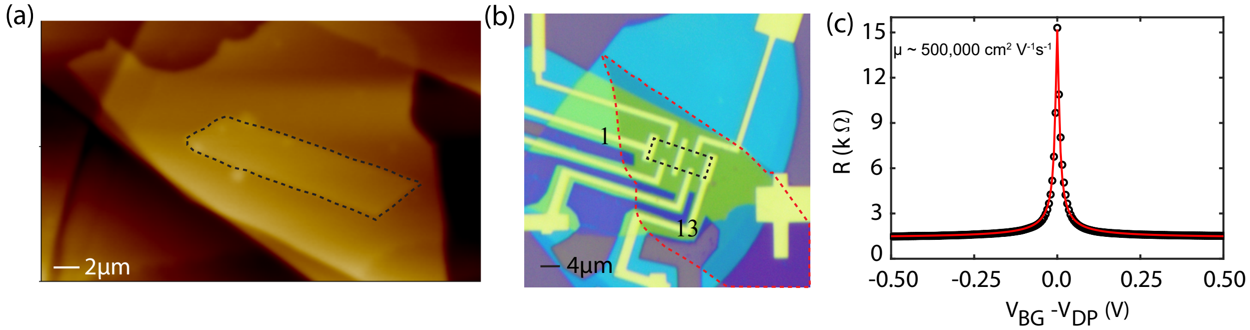

Supplementary Fig. 1: AFM image, optical image, and device characterization. (**a**) Atomic force microscopic (AFM) topography of the heterostructure. The graphene region is marked with dashed lines. The transport channel was defined in the bubble free region of the graphene flake. (**b**) Optical image of the final device structure. The region of graphene and the bottom graphite are marked by black and red dashed lines respectively. (**c**) The two-probe gate response measured between contact 1 and 13 (marked in Supplementary Fig. 1(b)), is plotted as a function of bottom graphite gate voltage at temperature 1.5 K. Open circles show the experimental data and the red curve is the fit of data in accordance with Supplementary Eq. (1). This fit gives a mobility of $\sim$ 500,000 $cm^2V^{-1}s^{-1}$. The high mobility of the device is necessary to observe fractional quantum Hall states. The charge inhomogeneity was found to be on the order of $\sim 2.3 \times 10^9 cm^{-2}$, which is one order of magnitude smaller than SiO_2 gated devices.

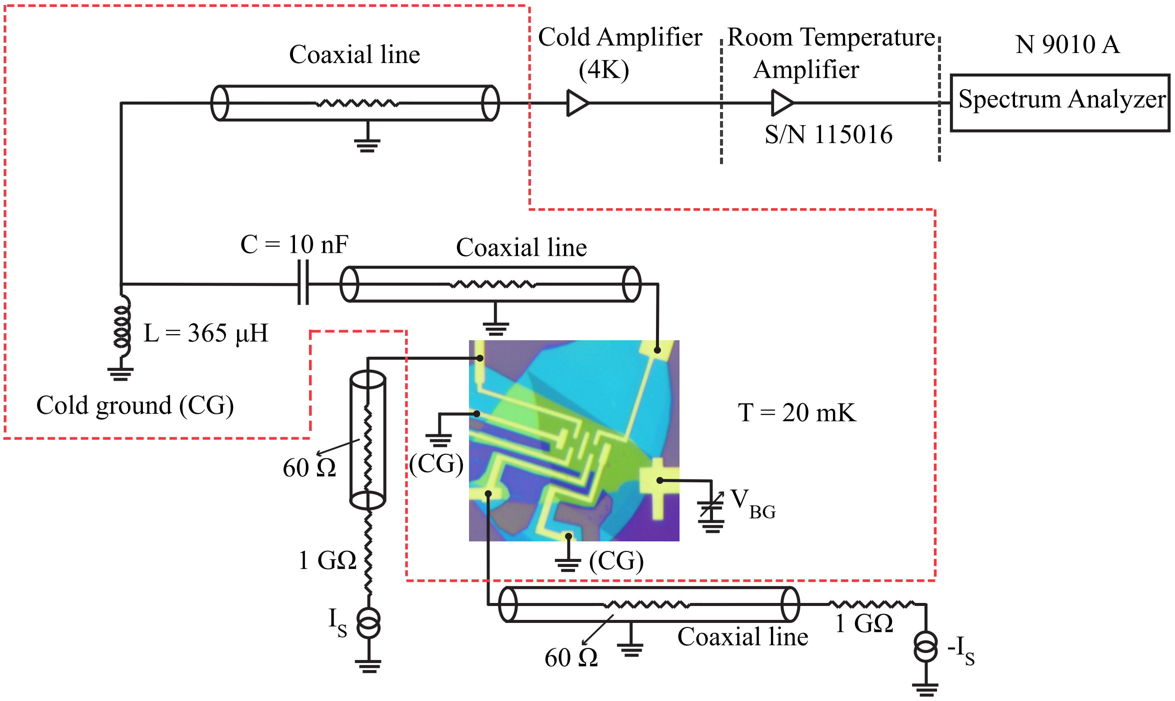

Supplementary Fig. 2: Experimental set-up for noise measurement. Schematic of the noise measurement set-up. The device was mounted on a chip carrier, which was connected to the home made cold finger fixed to the mixing chamber plate of dilution refrigerator. The ground contact (CG) pins are directly shorted to the cold finger to achieve the cold ground. The sample was current biased with currents $+I_S$ and $-I_S$ through two 1 GΩ resistors located at the top of dilution fridge. This was done in order to make the potential of the floating contact to be zero. To measure the temperature of the floating contact, we measure the current fluctuations. Measured current fluctuations are converted on chip into voltage fluctuations using the well defined quantum Hall (QH) resistance $R = h/\nu e^2$, where ν is the filling factor. This noise signal was amplified with a home made cryogenic voltage pre-amplifier, which was thermalized to 4K plate of dilution refrigerator. This pre-amplified signal was then amplified using a voltage amplifier placed at the top of the fridge at room temperature. After the second stage of amplification, the amplified signal was measured using a spectrum analyzer (N9010A). All the noise measurements were done using the bandwidth $\sim$ 30 kHz. The resonant L//C tank circuit was built using an inductor L of $\sim$ 365 μH made from a superconducting coil thermally anchored to the mixing chamber plate of the dilution refrigerator. A parallel capacitance C of $\sim$ 125 pF develops along the coaxial lines connecting the sample to the cryogenic pre-amplifier. A ceramic capacitance of 10 nF was introduced between sample and inductor to block the DC current along the measurement line.

Supplementary Note 2: Gain and Electron temperature calibration:

The gain of the amplification chain was estimated from temperature dependent Johnson-Nyquist noise (thermal noise)[6]. At zero impinging current, the equilibrium integrated voltage noise spectrum measured by the spectrum analyser is given as

$$S_V = g^2(4k_BTR + V_n^2 + i_n^2R^2)BW\,, \tag{2}$$

where g is the total gain of amplification chain, k_B the Boltzmann factor, T the temperature, R is the quantum resistance, V_n^2 and i_n^2 are the intrinsic voltage and current noises of the amplifier, and BW is the frequency bandwidth. At an integer quantum Hall plateau, any change in temperature of mixing chamber (MC) plate will only affect the first term in Supplementary Eq. (2), while all other terms are independent of T. If one plots $\frac{S_V}{BW}$ as a function of temperature, the slope of the linear curve will be equal to $4g^2k_BR$. Since at a quantum Hall plateau, the resistance R is exactly known, one can easily calculate the gain of the amplification chain and from the intercept, the intrinsic noises of the amplifier can be found. The gain g can then be calculated using the following equation

$$g = \sqrt{\Big(\frac{\partial\big(\frac{S_V}{BW}\big)}{\partial T}\Big)\Big(\frac{1}{4k_BR}\Big)}\,, \tag{3}$$

where $\left(\frac{\partial\left(\frac{S_V}{BW}\right)}{\partial T}\right)$ is the slope of the linear fit.

To find the electron temperature at zero impinging current, we measured the integrated voltage noise at resonance frequency for each bath temperature over time and then took the time average of the trace. The averaged integrated voltage noise is given by

$$S_V = g^2(4k_BTR + V_n^2 + i_n^2R^2)BW \tag{4}$$

Since we have already estimated the gain (from the slope) and the intrinsic noise of amplification chain (from the intercept), the corresponding electron temperature T_0 at base temperature of the mixing chamber plate can be found directly from the known value of the measured noise at zero bias. T_0 is given by

$$T_0 = \frac{\left(\left(\frac{S_V}{g^2BW}\right) - (V_n^2 + i_n^2R^2)\right)}{4k_BR} \tag{5}$$

The estimated electron temperature at several values of the bath temperature is shown in Supplementary Table 1.

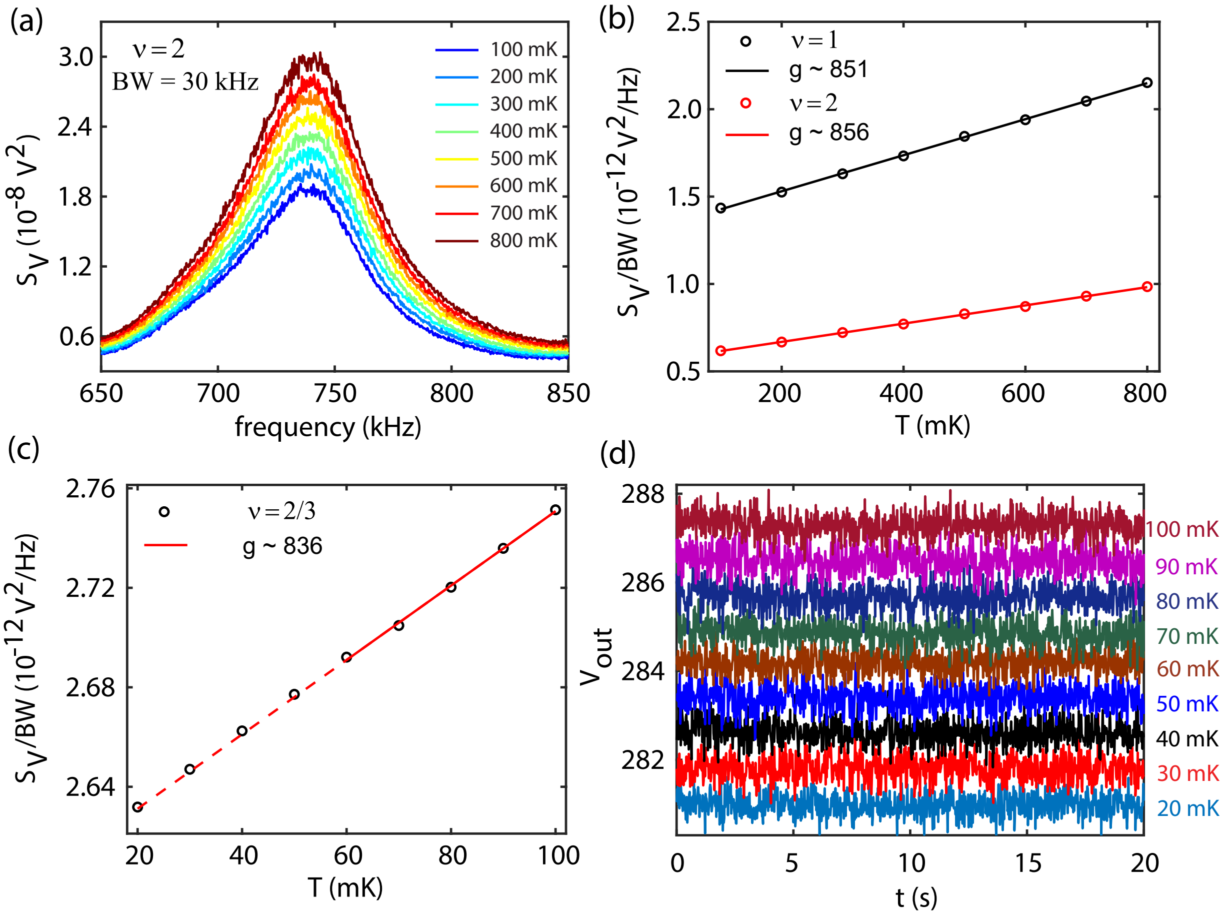

Supplementary Fig. 3: Gain of the amplification chain and electron temperature at zero impinging current. (**a**) The integrated voltage noise measured at zero bias is plotted as a function of frequency at different temperatures at $\nu = 2$. From these plots, the resonance frequency of the tank circuit was found to be ∼740 kHZ. (**b**) Open circles represent integrated noises divided by bandwidth (S_V/BW) at resonance frequency as a function of temperature for $\nu = 1$ (black) and $\nu = 2$ (red), respectively. Solid lines are the linear fit of these data points. Using Supplementary Eq. (3) and the slope information from these linear fits, the calculated gain was found to equal ∼851 ($\nu = 1$) and ∼856 ($\nu = 2$). (**c**) Symbols represent the plot of S_V/BW as a function of temperature at $\nu = 2/3$ at low temperatures. The solid red line is the linear fit of the data and from the slope of this line, the calculated gain was found to equal ∼836. (**d**) Each trace represents the measured output voltage V_{out} after the second stage of the amplification by the spectrum analyser at $\nu = 2/3$ for several values of the bath temperature shown by different colours. Each trace curve is the average of 200 scans. This measured output voltage is related to the voltage noise S_V via the relation $S_V = V_{out}^2$. The electron temperature was calculated using Supplementary Eq. (5).

Bath Temperature (mK)	20	25	30	35	40	45	50	55	60
Electron Temp (mK)	22.8	26.9	32.7	36.9	42.6	47.2	52.2	56.9	61.8

Supplementary Table 1: Bath temperature and corresponding electron temperature Table of bath temperature and corresponding estimated electron temperatures.

Supplementary Note 3: Joule heating and temperature (T_M) of the floating reservoir:

We have used two different configurations to achieve a hot metallic floating contact. In configuration 1, the metallic island remains at finite potential while in configuration 2, its potential is identically zero: the potential of the ground contacts. The noise data presented in the main manuscript is obtained using configuration 2. Here, we find the equations relating the dissipated power and the injected currents in both configurations.

Finite potential of floating contact. The current injection schematic is shown in Supplementary Fig. 4(a). In this configuration, the floating reservoir reaches a new equilibrium potential $V_M = \frac{I_S}{2\nu G_0}$ with the filling factor ν of graphene determined by V_{BG}. The potential of the S contact is $V_S = \frac{I_S}{\nu G_0}$. The power input to the floating reservoir is then $P_{in} = \frac{1}{2}(I_S V_S) = \frac{I_S^2}{2\nu G_0}$, where the pre-factor $\frac{1}{2}$ results due to the fact that equal power dissipates at the source and the floating reservoirs. Similarly, the outgoing power from the floating reservoir is $P_{out} = \frac{1}{2}(2 \times \frac{I_S}{2} V_M) = \frac{I_S^2}{4\nu G_0}$. Thus, the resulting injected power dissipation in the floating reservoir due to joule heating is $P_{in} - P_{out} = \frac{I_S^2}{4\nu G_0}$.

An alternative way to quantify the dissipation in floating contact is to calculate the power dissipation at the hot spots[7]. Whenever there is a change in the potential near contacts, hot spots will generate heat. There will be two hot spot located near the floating contact, two near cold ground contacts and one at the back of the source contacts as show in Supplementary Fig. 4(a). The power dissipated at the floating contact equals the sum of the power dissipated at the two hot spots formed near the floating contact. The half of the injected power from the source contact will drop at the back of the source contact and other half will be equally drop at four other hot spots, out of which two are formed near the floating contact and two near the cold ground contacts. The power dissipated near the floating contact is then $2 \times \left(\frac{1}{4}\left(\frac{I_S^2}{2\nu G_0}\right)\right) = \frac{I_S^2}{4\nu G_0}$.

Zero potential of floating contact. In this configuration, currents $+I_S$ and $-I_s$ are injected from two contacts as shown schematically in Supplementary Fig. 4(b). This leads to a zero potential of the floating contact. In this configuration, two hot spots form near the floating contact and two other are formed at the back of the source contacts as shown in Supplementary Fig. 4(b). Then, the dissipated power will be

$2 \times \left(\frac{I_S^2}{2\nu G_0}\right) = \frac{I_S^2}{\nu G_0}$.

Electron temperature of the floating contact. The resulting increase in the electron temperature ($T_M - T_0$) of the floating contact is determined from the generated excess thermal noise [8–12]: $S_I = 2G^* k_B(T_M - T_0)$ with $\frac{1}{G^*} = \frac{1}{G_L} + \frac{1}{G_R}$, where G_L and G_R are the conductance of left and right channel respectively. In our device structure, we have $\frac{1}{G^*} = \frac{1}{\nu G_0} + \frac{1}{\nu G_0}$, hence $S_I = \nu k_B(T_M - T_0)G_0$.

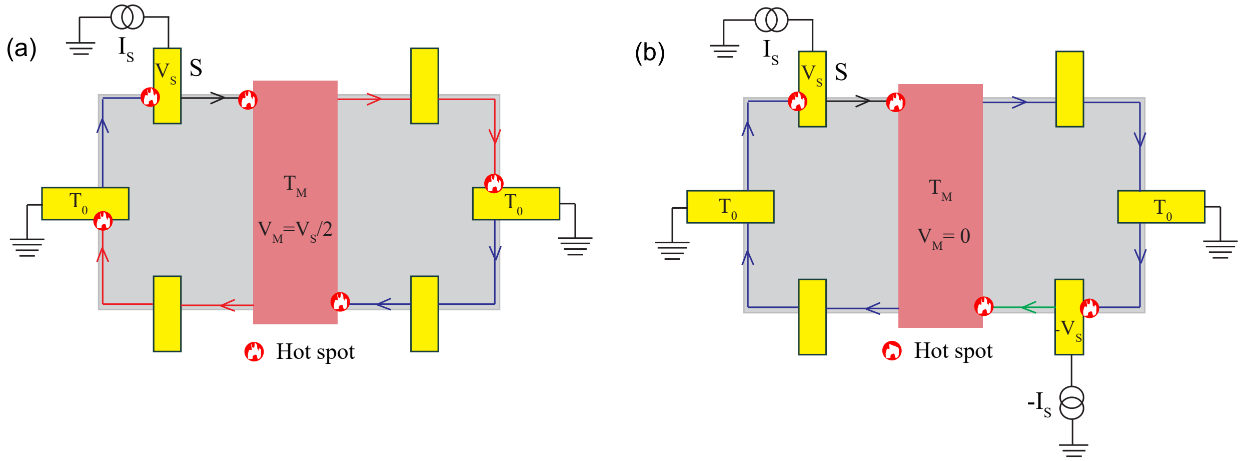

Supplementary Fig. 4: Current source configurations and corresponding hot spot positions. (a) Schematic of the current injection and hot spot positions in configuration 1. Here, the floating contact remains at finite potential. The current I_S injected from source contact S is carried along the quantum Hall edge channel to the floating contact. From the floating contact, the current splits into two equal parts, which propagate towards two cold grounds. In this scenario, the potential of the floating contact becomes half of the potential of the source contact. The power dissipation in this configuration is $J_Q = \frac{I_S^2}{4\nu G_0}$. **(b)** Configuration 2, which leads to zero potential of the floating contact. Current $+I_S$ and $-I_S$ are injected simultaneously from two diagonal contacts. In this configuration, no hot spots form near the cold ground contacts. The power dissipation in this configuration is $\frac{I_S^2}{\nu G_0}$.

Quasi-equilibrated electronic distribution in the metallic floating contact. The thermal conductance measurement technique employed in this work involves a floating metallic contact, which should act as a hot reservoir with a well defined quasi-equilibrated electronic distribution characterized by temperature T_M. In order to achieve this quasi-equilibrium regime, the heated electrons must dwell in metallic floating contact for a time sufficiently longer than the electron-electron interaction (thermalization) time scale. To estimate the dwell time inside the micron size floating metallic contact, we have followed the approach used by Jezouin, S. et al[8]. The dwell time is calculated using the equation $t_{dwell} = \frac{D_E \Omega h}{N}$[8, 13] , where D_E is the electronic density of states per unit volume per unit energy, Ω is the volume of micron-size floating contact, h is the Planck constant and N is the number of ideally coupled channels leaving the floating

contact. In our device, the floating contact has a volume $\Omega \approx 0.5\mu\text{m}^3$ and is mostly made of gold. By using the typical density of states for gold $D_E \simeq 1.14 \times 10^{47} J^{-1} m^{-3}$, we estimate the dwell time as $t_{dwell} \approx \frac{40\mu s}{N}$, which is much larger than the typical electron-electron interaction time, on the order of 10 ns for gold at a temperature down to few milli-kelvin, as experimentally demonstrated by F. Pierre et al [14]. Since the electron-electron interaction time remains orders of magnitude smaller than the dwell time, it firmly establishes that the electron energy distribution in the micron size metallic contact is a hot Fermi distribution function characterized by a temperature T_M.

Supplementary Note 4: Equipartitioning of the current and absence of reflection from the floating metallic contact.

Equipartitioning of the current. The equipartitioning of the injected current is crucial for the thermal conductance measurement. In other words, the bulk filling fractions on both side of the floating contact should be equal. This should be verified in order to rule out any possibility of bulk contributions as well as to ensure the validity of the dissipated power relation to the source current I_S. To verify this, we use a measurement configuration shown in Supplementary Fig. 5(a). Current is injected from the source contact S and the voltage is measured at contact S, R, and T. At the quantum Hall plateaus, the measured voltage at R and T contact is found to be half of the voltage measured at contact S, which establishes the equipartitioning of the current. In Supplementary Fig. 5(b), resistances measured at different contacts are plotted as functions of the back gate voltage. Here, the resistance is obtained by dividing the measured potential at different contacts by the injected current. Since the measured voltage at contact R and T is half of the voltage measured at contact S, the resistance value shown in Supplementary Fig. 5(b) at these contacts are found to be half of the original quantum resistance.

Absence of reflection coefficient from floating metallic contact. Since the electron temperature of the floating metallic contact is extracted by measuring the excess thermal noise, it is important to rule out the possibility of a significant reflection coefficient between the edge and the floating metallic contact. If present, such reflection can contribute to the measured excess noise. Although the exact equipartitioning of the current discussed in the previous section already establishes the absence of any detectable reflection coefficient in our device, to further emphasize this point, additional discussion is required, which should be explained for completeness. Supplementary Fig. 6(a) illustrates the situation of a constant current I injected from the source contact and a measurement of the voltage drops along the reflected and the transmitted paths. The corresponding resistances are defined as $R_R = V_R/I$ and $R_T = V_T/I$, where V_R and V_T are the voltages measured at contact R and T, respectively. Suppose that the reflection coefficient from the floating contact is r, a fraction Ir of the current (illustrated as the wiggly line in Supplementary Fig. 6(a)) will be reflected and will propagate along the bottom edge of the device due to the chirality of the quantum Hall edge. The transmitted current $I(1-r)$ will be equally split into two parts and propagate towards the two cold

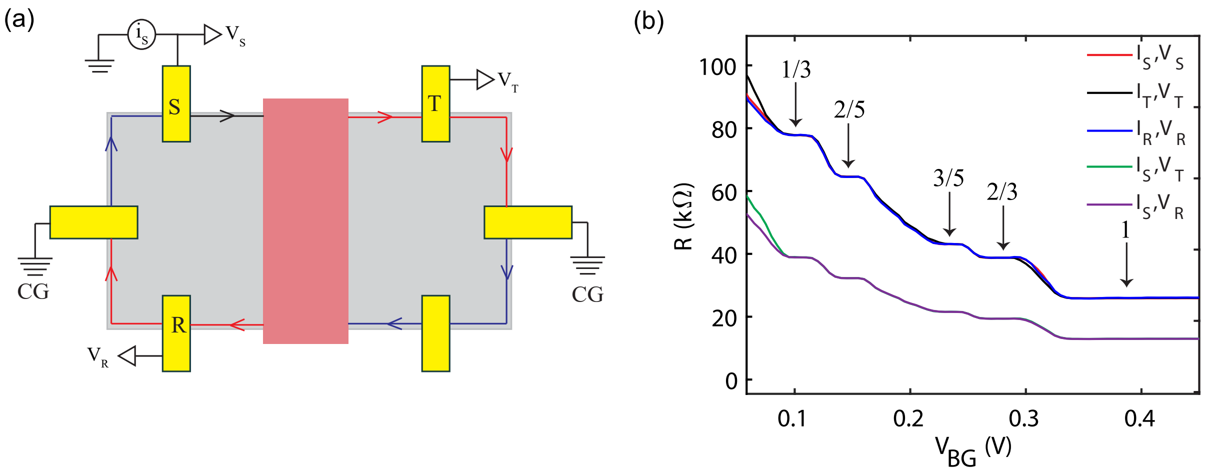

Supplementary Fig. 5: Equipartitioning of the current. **(a)** Schematic of the measurement configuration. Current i_S is injected from source contact S, and the voltage is measured at contacts S, R, and T. **(b)** The resistance R (V/i_S) measured at different contacts are plotted as functions of the gate voltage. Different colours represents traces taken at different contacts. In the legend, the subscript of the I and V shows the current injection and voltage probe contact respectively. At quantum Hall plateaus, the resistance curves taken at the contacts R (purple) and T (green) (injecting current at S) are lying on top of each other, which demonstrates that the filling on both side of the floating contact are equal. The halving of the magnitude of the resistance measured at these contacts establish that the injected current is equally split into two parts from the floating contact.

grounds. The total current flowing along the bottom edge on the left side is then $I_R = Ir + \frac{(1-r)I}{2} = \frac{(1+r)I}{2}$. The voltage measured at contact R will be given as $V_R = I_R \times R_q = \frac{(1+r)I}{2} \times R_q$ (R_q is the two-terminal resistance $\frac{h}{\nu e^2}$). As a result, the measured resistance at contact R will be $R_R = V_R/I = \frac{(1+r)}{2} \times R_q$. Similarly, the total current flowing along the top edge on the right hand side will be $I_T = \frac{(1-r)I}{2}$. The corresponding voltage measured at contact T will be $V_T = I_T \times R_q = \frac{(1-r)I}{2} \times R_q$ and the resistance will be $R_T = V_T/I = \frac{(1-r)}{2} \times R_q$. As a result, due to finite reflection coefficient of r, there must be a detectable difference between the measured resistances at contact R and T respectively. As can be seen from Supplementary Fig. 6(b), R_T and R_R are identical within the experimental resolution, and exactly half of the respective quantum resistance values of each plateau. This equality demonstrates that there is equal partitioning of the current in our device, and therefore rules out any detectable reflection from the floating contact. Note that even a 1% reflection implies a $\sim 800\Omega$ resistance difference between the two paths at the $\nu = 1/3$ plateau. This is not the case in our experiment.

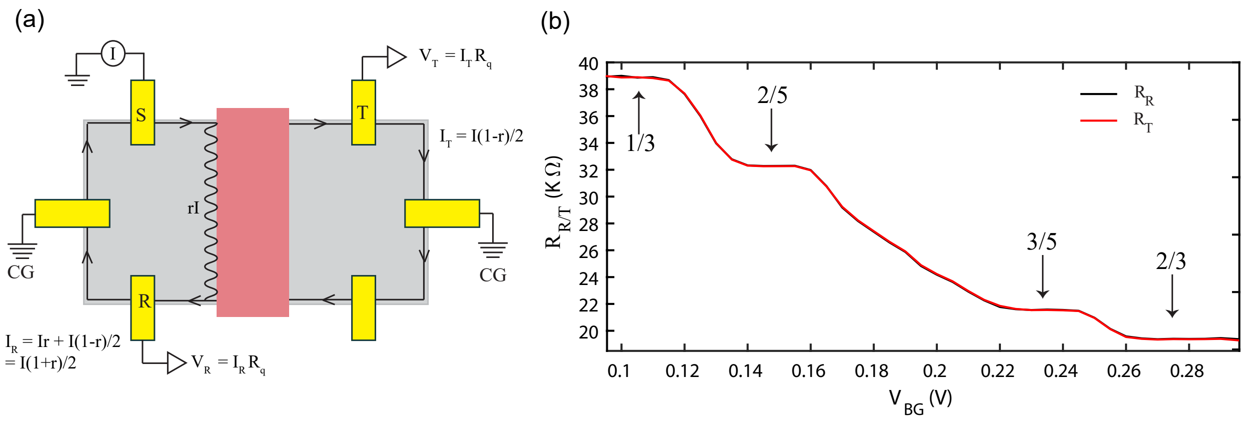

Supplementary Fig. 6: Absence of reflection coefficient from floating metallic contact. (a) Illustration of the current distribution in our device, accounting for edge-contact reflection with coefficient r. The wiggly line is shown for the illustrative purposes. It does not represent a microscopic picture of the current reflection. **(b)** Equipartition of the current on both sides for our device. The red (measured at contact T) and the black (measured at contact R) are exactly on top of each other at quantum Hall plateaus. This firmly establishes that the reflection coefficient from the metallic floating contact is at least non-detectable within the experimental resolution. Even a 1% reflection would result in a $\sim$ 800Ω resistance difference between the two paths at the $\nu = 1/3$ plateau.

Supplementary Note 5: Robustness of fractional plateaus.

In addition to equipartitioning of currents at the metallic contact, it is important that the fractional states remain robust at the maximum electron temperature ($\sim$ 100) mK reached in our measurements. To check that, we measure the transverse resistance in several configurations shown in Supplementary Fig. 5(a) at 20 mK, 60 mK, and 100 mK of bath temperatures. As can be seen from Supplementary Fig. 7(a,b), these measured resistance remain same, suggesting the robustness of the plateaus. Supplementary Fig. 7(b) also suggests that equipartitioning of the current is well established even at the maximum bath temperature of floating contact. In addition to the transverse resistance, we also measure the resistance in a configuration as shown in Supplementary Fig. 7(c), which encodes the longitudinal resistance at 20 mK and 100 mK of bath temperature. In this configuration, the current i_R is injected from contact R. The clockwise chirality ensures that the injected current terminates at the cold grounds at any QH plateaus. The resistance in this configuration, plotted in Supplementary Fig. 7(d), has the same properties as a longitudinal resistance: in the absence of bulk transport, the voltage V_S is determined by the equilibrium potential of the ground contact. The observation of the vanishing resistance plateaus at 20 mK (black) and 100 mK (red) further supports the robustness of the FQH states.

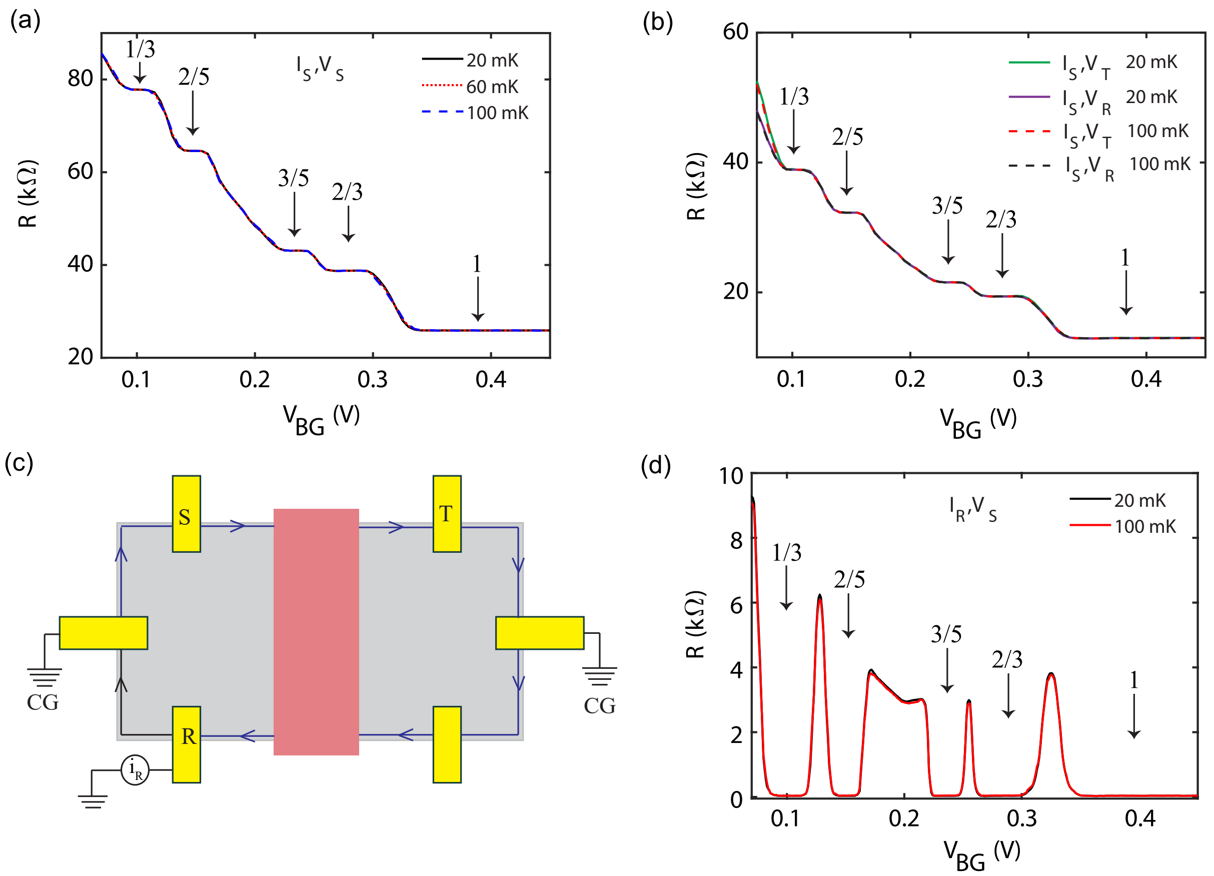

Supplementary Fig. 7: Robustness of quantum Hall states. **(a)** The resistance $R_S = V_S/i_S$ measured at contact S is plotted as functions of the gate voltage for 20, 60, and 100 mK bath temperatures.**(b)** The resistance $R_R(R_T)$ $(= V_R(V_T)/i_S)$ measured at contact $R(T)$ is plotted as functions of the gate voltage for 20 and 100 mK of bath temperature. **(c)** Schematic of the longitudinal resistance measurement configuration. Current i_R is injected from contact R, and the voltage is measured at contact S. The resistance measured in this configuration encodes the longitudinal resistance. **(d)** The resistance $R = V/i_R$ measured at contact S is plotted as function of the gate voltages. Black and red colours represent traces taken for 20 mK and 100 mK of the bath temperature, respectively. In the legend of all plots, the subscript of the I and V shows the current injection and voltage probe contact respectively. As can be seen from all resistance plots, the quantum Hall plateaus position and resistance values remain same at all values of the bath temperature, suggesting the robustness of the quantum Hall plateaus in our device.

Supplementary Note 6: Values of the electrical conductance without charge equilibration.

To estimate the electrical conductance values of hole-conjugate fractional quantum Hall states in the absence of charge equilibration along the propagation length for our device configuration, we follow a Landauer-

Büttiker approach [15]. In this calculation, we assume full charge equilibration at the Ohmic contacts including the floating metallic contacts. We calculate the electrical conductance at $\nu = 2/3$ and $\nu = 3/5$ edges, which are believed to host counter propagating bare charge modes in absent of the equilibration. The schematic of the device with contact number is shown in Supplementary Fig. S8.

The $\nu = 2/3$ edge: The multiprobe device geometry is shown in Supplementary Fig. 8(a). In the absence of charge equilibration, the edge structure of the $\nu = 2/3$ state consists of a downstream charge mode of charge e and an upstream mode of charge $-e/3$. For a multi-probe device, the net current flowing in i^{th} contact is given by

$$I_i = \sum_j (G_{j\leftarrow i}V_i - G_{i\leftarrow j}V_j), \tag{6}$$

where $G_{j\leftarrow i}$ is the conductance from the ith contact to the jth contact and V_i is the voltage of the ith contact. In matrix form, we then have

$$\begin{pmatrix} I_1 \\ I_2 \\ I_3 \\ I_4 \\ I_5 \\ I_6 \\ I_7 \end{pmatrix} = \frac{e^2}{h} \begin{pmatrix} 1+1/3 & -1/3 & 0 & 0 & 0 & 0 & -1 \\ -1 & 2(1+1/3) & -1/3 & 0 & -1 & -1/3 & 0 \\ 0 & -1 & 1+1/3 & -1/3 & 0 & 0 & 0 \\ 0 & 0 & -1 & 1+1/3 & -1/3 & 0 & 0 \\ 0 & -1/3 & 0 & -1 & 1+1/3 & 0 & 0 \\ 0 & -1 & 0 & 0 & 0 & 1+1/3 & -1/3 \\ -1/3 & -0 & 0 & 0 & 0 & -1 & 1+1/3 \end{pmatrix} \begin{pmatrix} V_1 \\ V_2 \\ V_3 \\ V_4 \\ V_5 \\ V_6 \\ V_7 \end{pmatrix}. \tag{7}$$

Contacts 4 and 7 are taken as grounded, and we may eliminate the corresponding entries from the matrix (7). After eliminating the rows and column associated with contacts 4 and 7, we get the reduced equation

$$\begin{pmatrix} I_1 \\ I_2 \\ I_3 \\ I_5 \\ I_6 \end{pmatrix} = \frac{e^2}{h} \begin{pmatrix} 1+1/3 & -1/3 & 0 & 0 & 0 \\ -1 & 2(1+1/3) & -1/3 & -1 & -1/3 \\ 0 & -1 & 1+1/3 & 0 & 0 \\ 0 & -1/3 & 0 & 1+1/3 & 0 \\ 0 & -1 & 0 & 0 & 1+1/3 \end{pmatrix} \begin{pmatrix} V_1 \\ V_2 \\ V_3 \\ V_5 \\ V_6 \end{pmatrix}. \tag{8}$$

Since current is injected only at contact 1, the current column reduces to

$$\begin{pmatrix} I_1 \\ I_2 \\ I_3 \\ I_5 \\ I_6 \end{pmatrix} = \begin{pmatrix} I \\ 0 \\ 0 \\ 0 \\ 0 \end{pmatrix}, \tag{9}$$

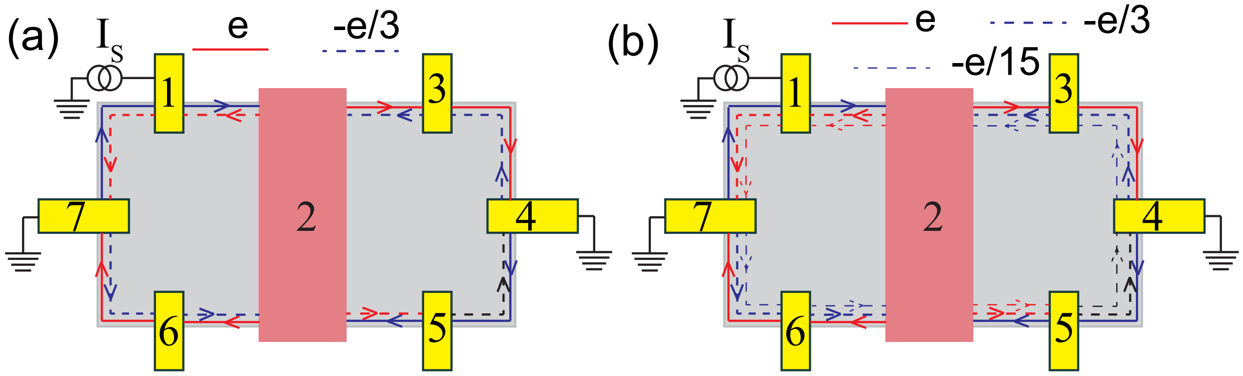

Supplementary Fig. 8: Schematic for the electrical conductance calculation using Landauer Büttiker Formalism. The contacts are marked by the numbers. **(a)** Bare edge modes for $2/3$ state. The charged mode with charge e is shown by solid line and charge mode with charge $e/3$ is shown by dashed line. **(b)** Bare edge modes for $3/5$ state. The charged mode with charge e is shown by solid line and charge mode with charge $e/3$ and $e/15$ are shown by thick and thin dashed line, respectively.

and we can solve for the voltages. We then find

$$\begin{pmatrix} V_1 \\ V_2 \\ V_3 \\ V_5 \\ V_6 \end{pmatrix} = I\frac{h}{e^2}\begin{pmatrix} 0.8625 \\ 0.4500 \\ 0.3375 \\ 0.1125 \\ 0.3375 \end{pmatrix}. \tag{10}$$

Voltage measured at contact 1 is therefore

$$V_1 = 0.8625 \times I\frac{h}{e^2} \tag{11}$$

and the conductance at source contact 1 is

$$G = \frac{I}{V_1} = 1.16\frac{e^2}{h}. \tag{12}$$

Similarly, the conductances at contacts 3 and 6 become

$$G = \frac{I}{V_3} = 2.96\frac{e^2}{h} \tag{13}$$

and, respectively,

$$G = \frac{I}{V_6} = 2.96\frac{e^2}{h}. \tag{14}$$

The $\nu = 3/5$ edge: The multiprobe device geometry is shown in Supplementary Fig. 8(b). In the absence of charge equilibration, the edge structure of the $\nu = 3/5$ state consists of one downstream charge mode of charge e and two upstream mode with charges $-e/3$ and $-e/15$. Following the same approach as for the $\nu = 2/3$ state, we here get the matrix equation (assuming contact 4 and 7 are grounded)

$$\begin{pmatrix} I_1 \\ I_2 \\ I_3 \\ I_5 \\ I_6 \end{pmatrix} = \frac{e^2}{h} \begin{pmatrix} 1+\frac{1}{3}+\frac{1}{15} & -\frac{1}{3}-\frac{1}{15} & 0 & 0 & 0 \\ -1 & 2(1+\frac{1}{3}+\frac{1}{15}) & -\frac{1}{3}-\frac{1}{15} & -1 & -\frac{1}{3}-\frac{1}{15} \\ 0 & -1 & 1+\frac{1}{3}+\frac{1}{15} & 0 & 0 \\ 0 & -\frac{1}{3}-\frac{1}{15} & 0 & 1+\frac{1}{3}+\frac{1}{15} & 0 \\ 0 & -1 & 0 & 0 & 1+\frac{1}{3}+\frac{1}{15} \end{pmatrix} \begin{pmatrix} V_1 \\ V_2 \\ V_3 \\ V_5 \\ V_6 \end{pmatrix}. \tag{15}$$

Current is only injected at contact 1, i.e.,

$$\begin{pmatrix} I_1 \\ I_2 \\ I_3 \\ I_5 \\ I_6 \end{pmatrix} = \begin{pmatrix} I \\ 0 \\ 0 \\ 0 \\ 0 \end{pmatrix}, \tag{16}$$

and the voltages become

$$\begin{pmatrix} V_1 \\ V_2 \\ V_3 \\ V_5 \\ V_6 \end{pmatrix} = I\frac{h}{e^2} \begin{pmatrix} 0.8374 \\ 0.4310 \\ 0.3079 \\ 0.1232 \\ 0.3079 \end{pmatrix}. \tag{17}$$

The voltage measured at contact 1 reads

$$V_1 = 0.8375 \times I\frac{h}{e^2}, \tag{18}$$

so that conductance at source contact 1 equals

$$G = \frac{I}{V_1} = 1.19\frac{e^2}{h}. \tag{19}$$

Similarly, the conductances at contacts 3 and 6 become

$$G = \frac{I}{V_3} = 3.25\frac{e^2}{h} \tag{20}$$

and, respectively,

$$G = \frac{I}{V_6} = 3.25\frac{e^2}{h}. \tag{21}$$

The conductances are summarized in Supplementary Table 2. The calculated conductances for the $\nu = 2/3$ edge was found to be $2.16\frac{e^2}{h}$ and $2.96\frac{e^2}{h}$ at source (S) and reflected/transmitted (R/T) contacts,

respectively. Similarly, for the $3/5$ edge, the calculated conductances were found to be $1.19\frac{e^2}{h}$ and $3.25\frac{e^2}{h}$ at source(S) and reflected/transmitted (R/T) contacts, respectively. However, in our experiment, the measured values of the conductance at source (I_S/V_S) and reflected/transmitted (I_S/V_R or I_S/V_T) contacts were found to be $0.67\frac{e^2}{h}$ and $1.33\frac{e^2}{h}$ for $\nu = 2/3$, and $0.60\frac{e^2}{h}$ and $1.20\frac{e^2}{h}$ for $\nu = 3/5$, respectively. These measured values suggest that the charge equilibration of the counter propagating edge modes along the propagation length is well established in our device.

Filling Factor(ν)	Calculated conductance in absence of charge equilibration (I/V_i) (in e^2/h)			Experimentaly measured conductance (I/V_i) (in e^2/h)		
	Source (1)	Reflected (2)	Transmitted (3)	Source (1)	Reflected (2)	Transmitted (3)
2/3	1.16	2.96	2.96	0.67	1.33	1.33
3/5	1.19	3.25	3.25	0.60	1.20	1.20

Supplementary Table 2: Comparison of calculated electrical conductances using the Landauer- Büttiker formalism and our measured conductances. The electrical conductances calculated for the hole-like states assuming no charge equilibration between counter-propagating edge channels are always much larger than the experimentally measured values. The measured values are instead in agreement with values expected for fully equilibrated edges.

Supplementary Note 7: Extraction of averaged noise data from raw data.

The excess thermal noise data presented in the main manuscript in Supplementary Fig. 2(a) and 2(d) and other excess thermal noise plots presented in this supplementary file is the averaged data of several experimental traces of raw data. This is explicitly demonstrated in Supplementary Fig. (9) for $\nu = 2$. The blue scan shown in Supplementary Fig. 9(a) is a single trace measured by the spectrum analyser. One can see that the data points are highly fluctuating and it is extremely difficult to do any quantitative analysis from this data. The red curve shown in Supplementary Fig. 9(b) is the average of $\sim$1000 such raw data traces. We follow the same procedure for all fractional fillings considered in this work. After getting this averaged data for the excess thermal noise, we extract J_Q and T_M from this data using the equations obtained in Supplementary Note 3. In Supplementary Fig. 9(b), the green curve shows the extracted data from Supplementary Fig. 9(a), while the black solid circles are the 9 point average of the corresponding green curve.

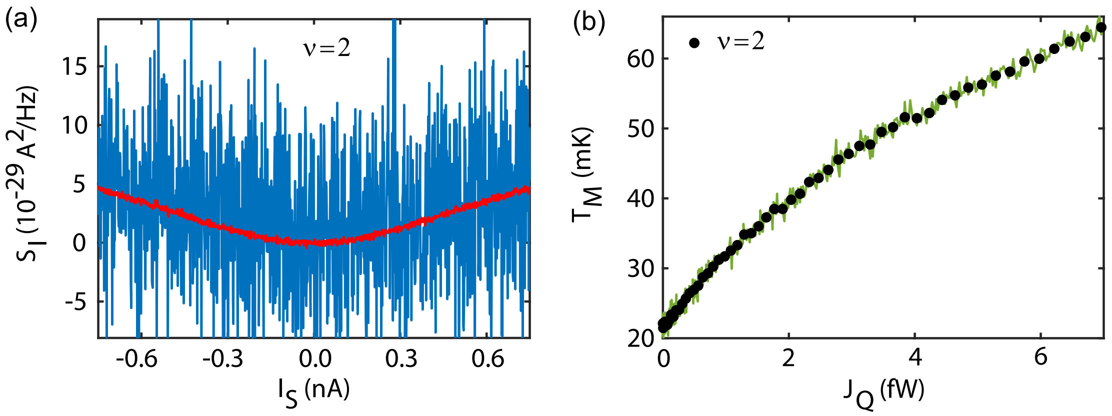

Supplementary Fig. 9: Extraction of average data from raw data (**a**) Excess noise for a single scan (blue) and the average of 1000 scans (red) at $\nu = 2$. (**b**) The solid green curve shows the data extracted directly from raw excess thermal noise data at $\nu = 2$. Filled black circles display 9 point averages of the corresponding raw data.

Supplementary Note 8: Extraction of the thermal conductance for the finite potential configuration of the floating contact.

In the main manuscript, all thermal noise data points shown are taken at zero potential of the floating contact. It is done by injecting currents $+I_S$ and $-I_S$ simultaneously from two contacts as shown schematically in Fig. 1(b) (main manuscript) and in Supplementary Fig. 4(b). We also repeat the thermal conductance measurement using conventional measurement configuration used in our previous works[16,17]. In this measurement configuration, the current is only injected from one contact as shown schematically in Supplementary Fig. 4(a), which leads to the finite potential of the floating contact at finite bias. The measured values of the thermal conductance in this configuration matches with the zero potential configuration of the floating contact shown in main manuscript.

Thermal conductance measurement of integer quantum Hall states: In this section, we discuss the measurement of the thermal conductance for integer fillings ν =1, 2 and 3, in the finite potential configuration of the floating contact. This is shown schematically in Supplementary Fig. 10(a). The current is injected from the contact S and the resulting excess thermal noise is measured at contact T. The excess thermal noise is plotted as function of the current I_S in Supplementary Fig. 10(b), 10(c), and 10(d) for ν =1, 2, and 3, respectively. The S_I and I_S axis from these plots are converted into T_M and J_Q, respectively, which is further plotted in Supplementary Fig. 10(e) for ν =1 (black), 2 (red), and 3 (blue). To extract the values of G_Q, J_Q is plotted as function of $T_M^2 - T_0^2$ for ν =1 (black), 2(red), and 3(blue). The solid circles represent the experimental data, while the solid curves are the linear fits of these data points with G_Q = 1.03, 1.99,

and 2.99 $\kappa_0 T$ for ν = 1, 2, and 3, respectively.

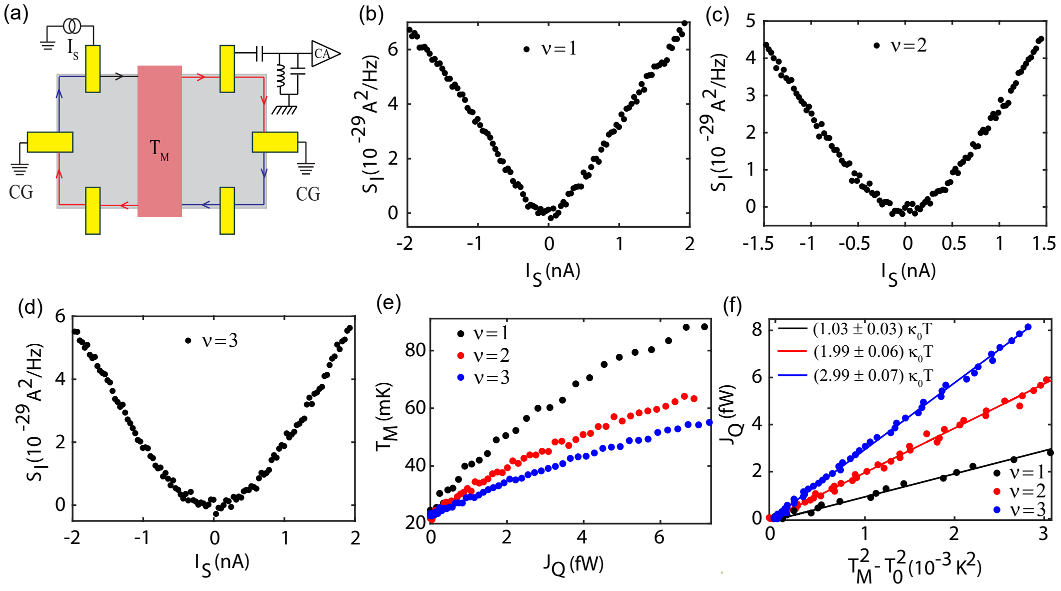

Supplementary Fig. 10: Thermal conductance measurement of integer quantum Hall states (**a**) Schematic of the measurement configuration. (**b**) Excess thermal noise S_I is plotted as a function of source current I_S at filling ν = 1 (b), 2 (c), and 3 (d). (**e**) Temperature T_M (extracted from the excess thermal noise shown in (b,c,d)) of floating contact is plotted as a function of dissipated power $P = J_Q$ (obtained using $P = \frac{I_S^2}{4\nu G_0}$ and the heat balance equation) for filling factors ν = 1 (black), 2 (red), and 3 (blue), respectively. Filled circles display the extracted temperature data using equation $S_I = \nu k_B(T_M - T_0)G_0$. (**f**) J_Q plotted as a function of $T_M^2 - T_0^2$. Filled circles display the data and the solid lines are the linear fits of these data points with $G_Q = 1.03$, 1.99, and 2.99 $\kappa_0 T$ for ν = 1, 2, and 3, respectively.

Thermal conductance measurement of fractional quantum Hall states: Similar to the integer quantum Hall states, the current is injected from the contact S and the resulting excess thermal noise is measured at contact T. The excess thermal noise is plotted as function of the current I_S in Supplementary Fig. 11(b), 11(c), 11(d), and 11(e) for ν =1/3, 2/5, 3/5, and 2/3, respectively. The S_I and I_S axis from these plots are converted into T_M and J_Q, respectively, which is further plotted for ν =1/3 (red) and 2/3 (black) in Supplementary Fig. 11(f), and for ν =2/5 (red) and 3/5 (black) in Supplementary Fig. 11(g). To extract the value of G_Q, J_Q is plotted as functions of $T_M^2 - T_0^2$ for ν =1/3 (red) and 2/3 (black) in Supplementary Fig. 11(h), and for ν =2/5 (red) and 3/5 (black) in Supplementary Fig. 11(i). The Filled circles represent the experimental data while the solid curves are the linear fits of these data points with G_Q = 1.02, 2.04, 2.02, and 3.05 $\kappa_0 T$ for ν = 1/3, 2/3, 2/5, and 3/5, respectively. All these data points were taken at bath temperature 30 mK.

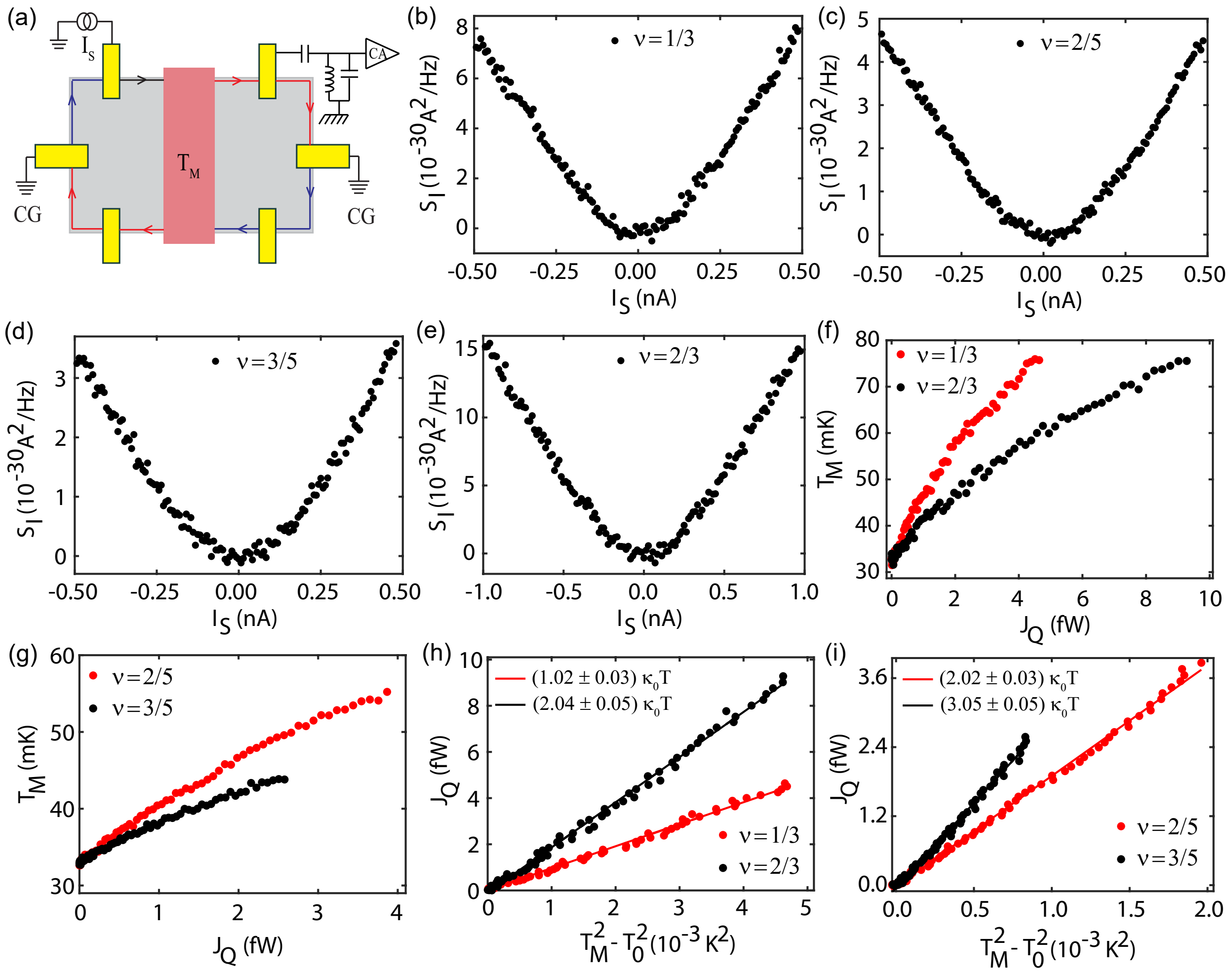

Supplementary Fig. 11: Thermal conductance measurement for fractional quantum Hall states (a) Schematic of the measurement configuration. (**b**) Excess thermal noise S_I is plotted as a function of the source current I_S at fillings ν = 1/3. (**c-e**) same as for panel (**b**) but for fillings 2/5, 3/5, 2/3. (**f, g**) Floating island temperature T_M (extracted from the excess thermal noise shown in (b,c,d,e)), plotted as a function of the dissipated power $P = J_Q$ (obtained using $P = \frac{I_S^2}{4\nu G_0}$ and the heat balance equation) for ν =1/3 (red) and 2/3 (black) in Supplementary Fig. 11(f), and for ν =2/5 (red) and 3/5 (black) in Supplementary Fig. 11(g). Filled circles display the extracted temperature data using equation $S_I = \nu k_B(T_M - T_0)G_0$. (**h, i**) J_Q plotted as a function of $T_M^2 - T_0^2$ for ν =1/3 (red) and 2/3 (black) in Supplementary Fig. 11(h), and for ν =2/5 (red) and 3/5 (black) in Supplementary Fig. 11(i). Filled circles display the data and the solid lines are the linear fits of these data points with G_Q = 1.02, 2.04, 2.02, and 3.05 $\kappa_0 T$ for ν = 1/3, 2/3, 2/5, and 3/5, respectively. All these data were taken at bath temperature of 30 mK. The G_Q of particle like (1/3, 2/5) states matches with expected value of $N_d\kappa_0 T$, while for hole like (2/3, 3/5) states, it matches with the non-equilibrated values of $(N_d + N_d)\kappa_0 T$. Here, N_d and N_u are the number of downstream and upstream modes, respectively.

Supplementary Note 9: Heat loss by electron-phonon Cooling.

The heat balance equation (1) in the main manuscript, contains, in addition to the electronic contribution, a mechanism of heat transfer via electron-phonon cooling (J_Q^{e-ph}). To estimate the contribution of J_Q^{e-ph}, we have subtracted the electronic contribution (J_Q^e) from the total heat current (J_Q),

$$J_Q^{e-ph} = J_Q - J_Q^e \,. \tag{22}$$

We have $J_Q^e = 0.5N\kappa_0(T_M^2 - T_0^2)$, where N is the total number of electronic channels leaving the floating contact. Usually, J_Q^{e-ph} has the functional form of $J_Q^{e-ph} = \beta(T_M^q - T_0^q)$. In our devices, J_Q^{e-ph} was found to be negligible below $\sim$ 100 mK at 20 mK of bath temperature. It can be seen from Supplementary Fig. S12(a) and S12(c) that the deviation from linearity happens beyond $\sim 100mK$ and $\sim 96mK$ for 20 mK (corresponding electron temperature $T_0 \sim$ 23 mK) and 50 mK (corresponding electron temperature $T_0 \sim 52$ mK) of bath temperatures, respectively. The power exponent (q) was found to be 5 in this case as shown in Supplementary Fig. S12(b,d). To avoid any contribution from electron-phonon cooling, we restricted our measurement to a maximum bath temperature (T_{bath}) of 60 mK, where T_M is increased to around 70-80 mK in order to fit a sufficient range of data for the G_Q extraction (Fig. 3 of the main manuscript). We could have increased the T_{bath} to further 5 to 10 mK, but this will not affect the central result of the current work as for $\nu = 3/5$ we have already seen the thermal conductance plateau for the equilibrated regime, and for $\nu = 2/3$, the asymptotic decay to $0.5\kappa_0 T$ for the diffusive nature. It should be mentioned that q was found to be varying in the range between 4 and 6 in our earlier work[16,17] and elsewhere[18]. Although the deviation from linearity shown in Supplementary Fig. S12(a) is consistent with a heat loss due to electron-phonon cooling, other possible mechanisms of heat loss can not be ruled out completely.

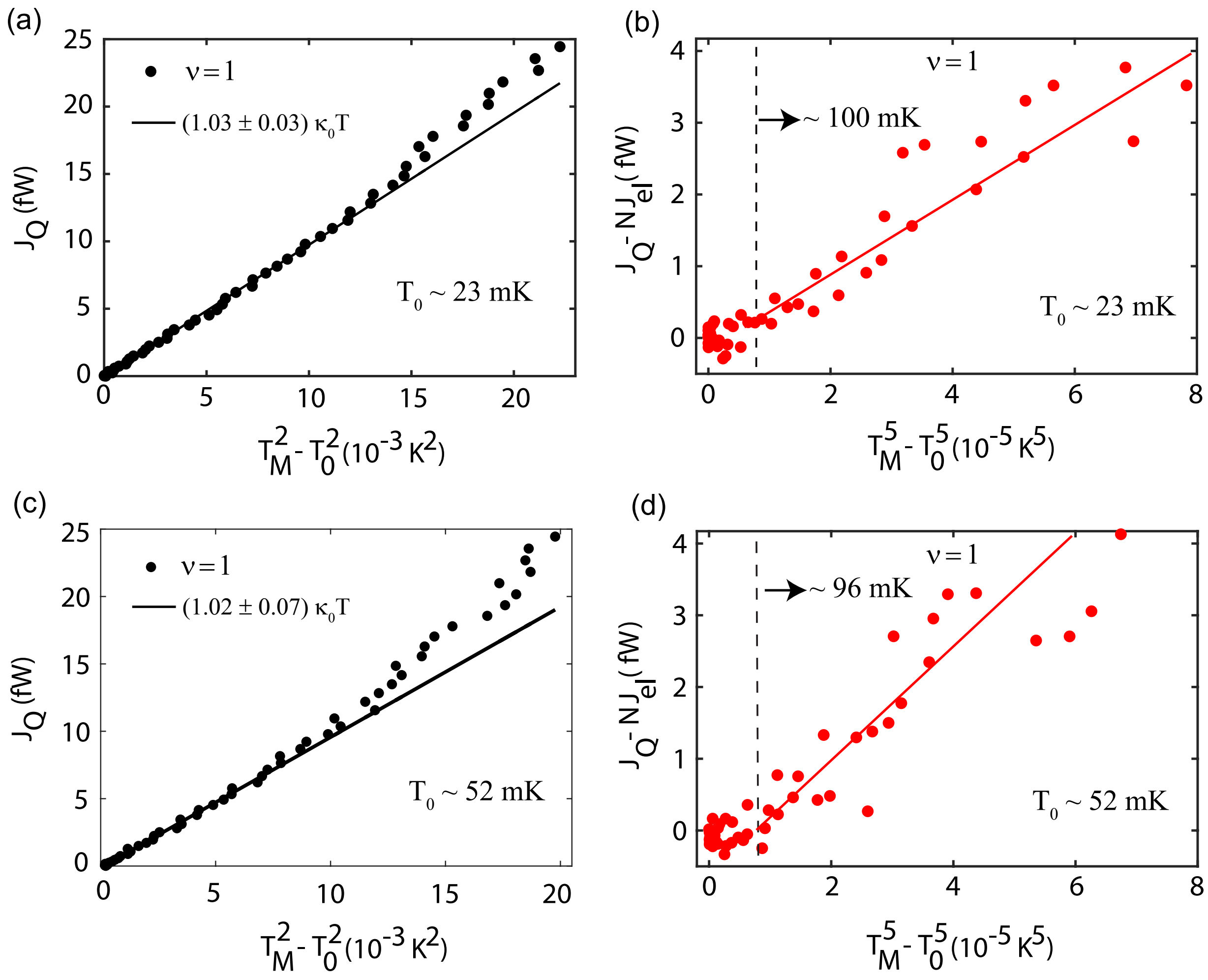

Supplementary Fig. 12: Heat loss by electron-phonon cooling. **(a)** J_Q plotted as function of $(T_M^2 - T_0^2)$ for $\nu = 1$ at 20 mK (corresponding electron temperature $T_0 \sim 23$ mK) of bath temperature. The solid circles represents the experimental data while the solid curve shows the theoretical line corresponding to the electronic contribution of heat flow. It can be seen that the experimental data points start deviating from the theoretical line beyond $\sim$ 15 fW. This deviation is attributed to heat losses by electron-phonon cooling. **(b)** Solid circles display the electron-phonon contribution of the heat loss (J_Q^{e-ph}) as a function of $(T_M^5 - T_0^5)$ for $\nu = 1$. The solid line is a linear fit with slope $\beta \sim 0.053 nW/K^5$. The contribution of J_Q^{e-ph} is only seen beyond $\sim$ 100 mK, as indicated by the vertical dashed line. **(c)** Similar plot as **(a)**, but now plotted at 50 mK (corresponding electron temperature $T_0 \sim 52$ mK) of bath temperature. **(d)** The contribution of J_Q^{e-ph} becomes significant beyond $\sim$ 96 mK, as indicated by the vertical dashed line.

Supplementary Note 10: Contact resistance and source noise.

The finite contact resistance of the source contact can create additional unwanted noise. To estimate this noise, we first need to have some estimate of the contact resistance. In our case, the contact resistance was extracted from the low-frequency resistance data. As shown in Supplementary Fig. 5(a), a current i_S was injected at source contact and the voltage was measured at the same contact. This voltage probe measures the voltage $i_S.(R_0 + R_L + R_C)$ with R_0 the quantum Hall resistance, R_C the contact resistance, and R_L the line resistance. The measured resistance will then be $R_0 + R_L + R_C$. We have measured the line resistance R_L to 265 Ω separately. After subtracting the line resistance, the conductance will be equal to $\frac{1}{R_0+R_C}$. Hence, the transmittance t will be given by $t = \frac{R_0}{R_0+R_C}$. Once the transmittance is known, one can determine the source noise $2eI(1-t)$. Since the amplifier is situated in the path of right moving edge channels in one arm of the device, it will measure only part of the generated source noise. In particular, for our device configuration, the amplifier will always measure only a $\frac{1}{4}$th of the source noise. The estimated contact resistance, transmittance and the source noise is shown in Supplementary Table 3. The reflection coefficient was always less than 0.25% for all fractional filling factors. The source noise measured by the amplifier would therefore be at least 2-3 orders of magnitude smaller than the measured excess thermal noise.

Filling Factor(ν)	Measured resistance (R_0+ R_L+ R_C) (Ω)	Line resistance R_L (Ω)	Contact Resistance (R_0+ R_L+ R_C) - R_L - R_0 in (Ω)	Trans-mittance (t)	Source Noise / 4 ($10^{-30} A^2$/ Hz) at
1/3	77888	265	184	0.9976	0.016 @ I_{max}= 0.25 nA
2/5	64866	265	68	0.9989	0.004 @ I_{max}= 0.25 nA
3/5	43353	265	66	0.9985	0.006 @ I_{max}= 0.25 nA
2/3	39029	265	45	0.9988	0.008 @ I_{max}= 0.25 nA

Supplementary Table 3: Contact resistance and source noise. Measured and estimated values for the contact resistance, the transmittance, and the maximum source noise for fractional filling factors.

Supplementary Note 11: Temperature dependence of the thermal conductance for the integer quantum Hall state

To further strength our observation of a temperature induced crossover of the thermal conductance for hole-like states, we also measure the thermal conductance of an integer quantum Hall state ($\nu = 1$) at increased bath temperature (50 mK). In Supplementary Fig. 13, we plot J_Q as a function of $T_M^2 - T_0^2$ at 20 mK (black) and 50 mK (red) bath temperatures. The thermal conductance value is the same at both temperatures, as expected theoretically for the integer quantum Hall state, where only downstream edge modes exist.

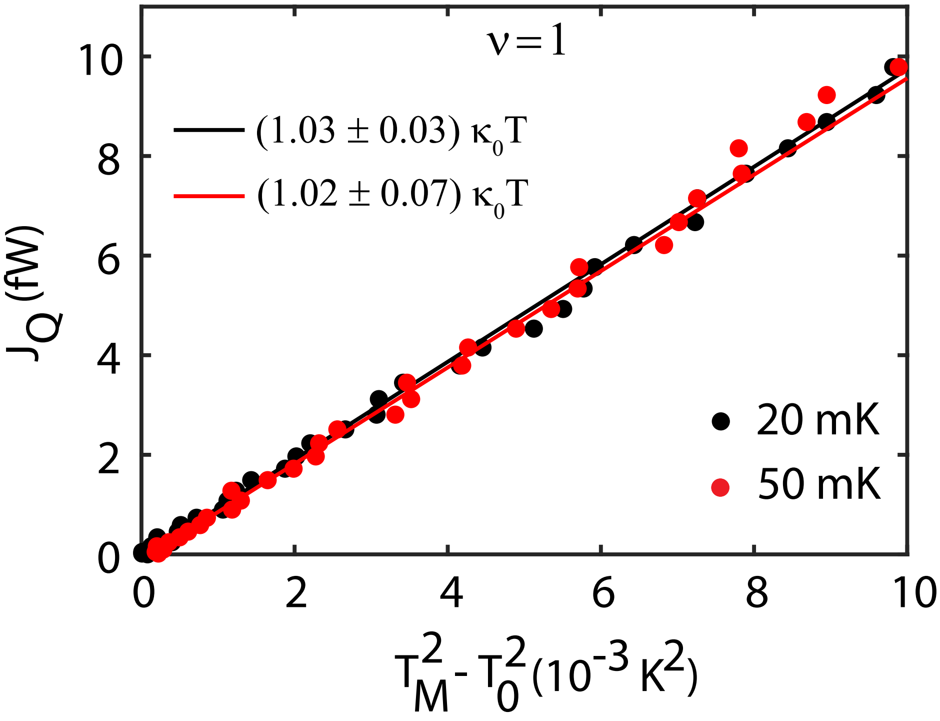

Supplementary Fig. 13: Temperature dependence of thermal conductance of the integer quantum Hall state. J_Q (solid circles) is plotted vs $(T_M^2 - T_0^2)$ at $\nu = 1$ for bath temperatures 20 mK (black) and 50 mK (red). Solid black and red lines are linear fits with $G_Q = 1.03\kappa_0 T$ and $1.02\kappa_0 T$ for 20 mK and 50 mK of bath temperature, respectively. The thermal conductance remains the same at both temperatures, as expected for the integer quantum Hall edge structure with a single downstream mode.

Supplementary Note 12: Discussion on edge reconstruction

In quantum Hall physics, the issue of edge reconstruction may raise concerns over the validity of the bulk-edge correspondence principle. Specifically, it has been proposed that edge reconstruction might occur in various QH states[19,20]. Here, we argue that our present work of measuring the thermal conductance is an excellent tool to probe whether edge reconstruction actually takes place. For example, for the $\nu = 1/3$ state, the edge reconstruction would increase the total number of modes from 1 to 3. This would lead to a crossover from $G_Q = 3\kappa_0 T$ at lower temperatures (non-equilibrated regime) to $G_Q = 1\kappa_0 T$ at higher temperatures (equilibrated regime), which is very different from what we observe ($G_Q = 1\kappa_0 T$ at all temperatures). Similarly, for the $\nu = 2/3$ state, edge reconstruction would increase the number of modes from 2 to 4. This would mean that, at low-temperatures (the non-equilibrated regime), the value of the thermal conductance will be; $G_Q = 4\kappa_0 T$. By the same token, for the $\nu = 3/5$ edge (proposed to have 3 edge channels), non-equilibrated heat transport would give rise to $G_Q = 5\kappa_0 T$. Clearly, no traces of these values are observed in our measurements. These results point strongly towards the absence of edge reconstruction in our device.

Edge reconstruction is a result of competition between the confinement potential that keeps the electrons in the interior of the sample, and Coulomb repulsion that tends to spread out the electron density. It has been theoretically predicted by Zi-Xiang Hu et al [21] that edge reconstruction in graphene for Laughlin edges, can be avoided, provided the separation between the graphene channels to the screening layer (metallic gate d) and the magnetic length scale ($l_B = \sqrt{\frac{\hbar}{eB}}$) are comparable ($0.5 l_B \leq d \leq 1.5 l_B$). In our device, the hBN thickness (25 nm) separating the graphene and the bottom graphite gate is very close to the limit of this criterion, we can in principle, expect that edge reconstruction does not take place in our device. Again, this picture is experimentally verified by our thermal conductance measurements. We believe that it is also in well accordance with the STM experiment performed for monolayer graphene supported on graphite [22]. In this work, the authors did not observe any sign of edge reconstruction for integer quantum Hall states. In addition, it is worth to mention here that edge reconstruction in fact has been reported in a local scanning gate microscope experiment[23]. There, the hBN encapsulated monolayer graphene device was supported on a SiO_2/Si substrate, and hence the graphene channel was separated by a ≈ 300 nm SiO_2 layer from the metallic gate (highly doped Si). This distance is much larger than the theoretical limit from Ref. [21]. Together, these reports suggest that edge reconstruction can be avoided in graphene if the confining potential is sharp enough, consistent with our findings.

Supplementary References

1. Pizzocchero, F. *et al.* The hot pick-up technique for batch assembly of van der waals heterostructures. *Nature communications* **7**, 11894 (2016).

2. Venugopal, A. *et al.* Effective mobility of single-layer graphene transistors as a function of channel dimensions. *Journal of Applied Physics* **109**, 104511 (2011).

3. Kumar, C. *et al.* Localization physics in graphene moiré superlattices. *Phys. Rev. B* **98**, 155408 (2018).

4. Kumar, C., Srivastav, S. K. & Das, A. Equilibration of quantum hall edges in symmetry-broken bilayer graphene. *Phys. Rev. B* **98**, 155421 (2018).

5. Kuiri, M. *et al.* Enhanced electron-phonon coupling in doubly aligned hexagonal boron nitride bilayer graphene heterostructure. *Phys. Rev. B* **103**, 115419 (2021).

6. Choi, B.-R. *et al.* Shot-noise and conductance measurements of transparent superconductor/two-dimensional electron gas junctions. *Physical Review B* **72**, 024501 (2005).

7. Spånslätt, C., Park, J., Gefen, Y. & Mirlin, A. D. Topological classification of shot noise on fractional quantum hall edges. *Phys. Rev. Lett.* **123**, 137701 (2019).

8. Jezouin, S. *et al.* Quantum limit of heat flow across a single electronic channel. *Science* **342**, 601–604 (2013).

9. Sivan, U. & Imry, Y. Multichannel landauer formula for thermoelectric transport with application to thermopower near the mobility edge. *Physical review B* **33**, 551 (1986).

10. Jiang, J.-H. & Imry, Y. Linear and nonlinear mesoscopic thermoelectric transport with coupling with heat baths. *Comptes Rendus Physique* **17**, 1047–1059 (2016).

11. Beenakker, C. & Büttiker, M. Suppression of shot noise in metallic diffusive conductors. *Physical Review B* **46**, 1889 (1992).

12. Blanter, Y. M. & Sukhorukov, E. Semiclassical theory of conductance and noise in open chaotic cavities. *Physical review letters* **84**, 1280 (2000).

13. Brouwer, P. & Büttiker, M. Charge-relaxation and dwell time in the fluctuating admittance of a chaotic cavity. *EPL (Europhysics Letters)* **37**, 441 (1997).

14. Pierre, F. *et al.* Dephasing of electrons in mesoscopic metal wires. *Phys. Rev. B* **68**, 085413 (2003).

15. Büttiker, M. Absence of backscattering in the quantum hall effect in multiprobe conductors. *Physical Review B* **38**, 9375 (1988).

16. Srivastav, S. K. *et al.* Universal quantized thermal conductance in graphene. *Science Advances* **5** (2019).

17. Srivastav, S. K. *et al.* Vanishing thermal equilibration for hole-conjugate fractional quantum hall states in graphene. *Phys. Rev. Lett.* **126**, 216803 (2021).

18. Sivre, E. *et al.* Heat coulomb blockade of one ballistic channel. *Nature Physics* **14**, 145 (2018).

19. Wan, X., Yang, K. & Rezayi, E. H. Reconstruction of fractional quantum hall edges. *Phys. Rev. Lett.* **88**, 056802 (2002).

20. Wan, X., Rezayi, E. H. & Yang, K. Edge reconstruction in the fractional quantum hall regime. *Phys. Rev. B* **68**, 125307 (2003).

21. Hu, Z.-X., Bhatt, R. N., Wan, X. & Yang, K. Realizing universal edge properties in graphene fractional quantum hall liquids. *Phys. Rev. Lett.* **107**, 236806 (2011).

22. Li, G., Luican-Mayer, A., Abanin, D., Levitov, L. & Andrei, E. Y. Evolution of landau levels into edge states in graphene. *Nature communications* **4**, 1–7 (2013).

23. Marguerite, A. *et al.* Imaging work and dissipation in the quantum hall state in graphene. *Nature* **575**, 628–633 (2019).
